# Effects of Stochastic Resonance Whole-Body Vibration in Individuals with Unilateral Brain Lesion: A Single-Blind Randomized Controlled Trial: Whole-Body Vibration and Neuromuscular Function

**DOI:** 10.1155/2018/9319258

**Published:** 2018-08-01

**Authors:** Kaspar Herren, Stefan Schmid, Slavko Rogan, Lorenz Radlinger

**Affiliations:** ^1^Department of Physiotherapy, Bern University Hospital, Inselspital Bern, Switzerland; ^2^SRO AG, Hospital of Langenthal, Langenthal, Switzerland; ^3^Department of Health Professions, Bern University of Applied Sciences, Bern, Switzerland; ^4^Academy of Physiotherapy and Training Education, Grenzach-Wyhlen, Germany

## Abstract

**Introduction:**

Stochastic resonance whole-body vibration (SR-WBV) devices are promising sensorimotor interventions to address muscle weakness and to improve balance and mobility particularly in the elderly. However, it remains inconclusive whether individuals with stroke or traumatic brain injury (TBI) can profit from this method. The aim of this prospective single-blind randomized controlled trial was to investigate the effects of SR-WBV on muscle strength as well as gait and balance performance in this population.

**Methods:**

Forty-eight individuals with stroke or TBI were randomly allocated to an experimental and a sham group. Participants were exposed daily to 5 consecutives 1-minute SR-WBV sessions, whereas the experimental group trained in a standing position with 5 Hz and the sham group in a seated position with 1 Hz. Isometric muscle strength properties of the paretic knee extensor muscles as well as balance and gait performance were measured at baseline, after the first session and after two weeks of SR-WBV.

**Results:**

Both groups showed short- and long-term effects in gait performance. However, no between-group effects could be found at the three measurement points.

**Discussion:**

Complementary SR-WBV showed no beneficial effects immediately after the intervention and after two weeks of conventional rehabilitation therapy. Future research is needed to identify the potential efficacy of SR-WBV in individuals with stroke and TBI using shorter and less exhausting test procedures and a generally prolonged intervention time.

## 1. Introduction

Stroke was reported to be the largest cause of complex disability in adults and traumatic brain injury (TBI) is the most common cause of long-term disability and death among young adults [1–5]. Both conditions represent an enormous socioeconomic and healthcare burden [5–7]. Individuals with stroke or TBI suffer from reduced muscle strength, spasticity, sensorimotor deficits, contractures, impaired balance resulting in gait disorders, and therefore reduced independence in everyday life [8–13]. It was shown that individuals with stroke walking at 0.25 m/s (SD 0.1) only achieve household ambulation, whereas only 18% are able to walk at 0.80 m/s (SD 0.15) after rehabilitation, a gait speed needed for community ambulation [10, 14–16]. In addition, they were at a four times higher risk of falling and a ten times higher risk of hip fractures compared to healthy individuals [14]. For these reasons, many of the stroke or TBI survivors are not anymore able to participate in their premorbid social and daily life [8, 17].

A major aim of neurological rehabilitation is to improve muscle function, balance, and gait performance, with improving walking ability being one of the most often stated goals by individuals with stroke [18, 19]. Lower extremity strength and balance performance appear to be interrelated as well as directly correlated with gait performance in individuals with stroke and TBI, whereby decreases in these parameters are reported to be important risk factors for falls in these populations [9, 20–27]. The evident sensorimotor impairments can be treated with a conventional multidisciplinary rehabilitation approach, which was recently successfully supplemented by robotic devices, virtual reality, treadmill training, electrical stimulation, or whole-body vibration (WBV) training [15, 28–31].

Vibration plates that are frequently used in elderly population [30, 33, 34] generate either sinusoidal vertical vibration with a frequency between 30 and 60 Hz and amplitude of 0-12 mm or side alternating sinusoidal vibrations with a frequency of 12-30 Hz and an amplitude of 0-12 mm [30, 33]. Huang et al. [35] postulated WBV amplitude, frequency, body postures, and their interactions significantly influenced the vibration transmissibility and signal purity among person with chronic stroke. The transmissibility decreased with increased frequency, increased amplitude, or increased knee flexion angle. The average vibration intensity measured was up to 4.94 g and the transmissibility ratio was 0.04-0.30 and the vibration intensity was 0.11-0.60 g.

The evidence on the treatment effects of sinusoidal WBV (SS-WBV) in individuals with stroke, however, is somewhat contradictory. Whereas some studies showed beneficial short- and long-term effects on gait and balance performance as well as mobility, trunk stability, muscle strength, and muscle tone [36–42], others reported no benefits or even adverse effects of SS-WBV compared to conventional exercise therapy [43–50]. In addition, no studies were found investigating the effects of SS-WBV on the impairments of individuals with TBI.

The physiological mechanisms responsible for the effects of SS-WBV are based on different theories. On the one side, it was postulated that SS-WBV causes changes in the length of the muscle-tendon complex, which, as a consequence, stimulates muscle spindles leading to increased reflexive activation of the alpha-motor units [32, 51]. On the other side, an increase of intramuscular temperature or a postactivation potentiation of the muscle twitch response was hypothesized to lead to an acute enhancement of muscle power [52, 53]. Moreover, changes in thixotropic properties of the vibrated muscles, enhanced hormonal secretion of testosterone, cortisol, and growth hormones, and even placebo effects might be responsible for effects such as strength increase [46, 54–56].

In contrast to SS-WBV platforms, the Zeptor med® vibrates randomly (stochastic) in three different planes with frequencies of 1-12 Hz and an amplitude of 3 mm [34, 57, 58]. In contrast to sinusoidal signals, stochastic stimuli are known to efficiently affect the membrane potential of nerve cells, resulting in an activation of the neuromuscular systems already at low intensities [34]. However, no studies are available evaluating stochastic resonance WBV (SR-WBV) as a treatment option for individuals with stroke or TBI. In general, any of the aforementioned forms of WBV can be recommended as a safe additional training intervention with very rare and normally harmless side effects such as tingling sensations in the legs, muscle soreness, fatigue, or mild dizziness [50, 59]. The physical strain of WBV is low and the training is not time consuming and therefore potentially useful for many different complaints of young and even frail elderly people [28, 60–62]. For these reasons, the aims of the current study were to investigate whether a complementary SR-WBV intervention has beneficial short- and long-term effects on isometric muscle strength properties, balance, and gait performance in individuals with an acute unilateral brain lesion due to stroke or TBI.

## 2. Methods

### 2.1. Study Design

This study was conducted as a single-center, single-blind randomized controlled trial. The study protocol was approved by the ethics committee of the Cantone of Bern, Switzerland (Reference no. 225/08).

### 2.2. Participants

Study participants were consecutively recruited among individuals with a first-ever stroke or TBI that were hospitalized at the Neurology Department of the Bern University Hospital between June 2010 and October 2014. Inclusion criteria were clinical diagnosis of an acute (less than 3 months but more than 8 days after onset) first-ever unilateral brain lesion by means of stroke or TBI, aged between 18 and 80 years, presence of balance and gait disorders but the ability to stand still and to walk 10 meters without assistance, and sufficient cognitive and linguistic skills to understand the test and therapy instructions. Individuals were excluded in case of documented comorbidities such as Parkinson's disease, polyneuropathy, severe uncorrected visual impairments or alcohol abuse, and complaints defined as contraindications for the intervention [63–65]. All participants provided written informed consent before inclusion.

The allocation to an experimental or a sham group as well as the determination of the order of the biomechanical measurements was concealed and conducted separately for the individuals with stroke or TBI using computer-generated 4-block randomization schemes. For each wave of participants, the prepared sealed opaque envelopes were randomly allocated by an administrative assistant not associated with the study. Participants, investigators, and statistician were blinded concerning assignment to interventions, whereas therapists could not be blinded.

### 2.3. Measurement Procedures

After inclusion, the participants' performance in activities of daily life, injury severity, and self-perceived fear of falling was assessed using the Extended Barthel Index (EBI), the National Institute of Health Stroke Scale (NIHSS), and the Falls Efficacy Scale International (FES-I) [66–69]. Subsequently, the participants' affected leg was equipped with bipolar surface electrodes (Ambu Blue Sensor N, Ambu A/S, Ballerup, Denmark) for the derivation of the electromyographic (EMG) activity of the muscles vastus medialis (VM), vastus lateralis (VL), tibialis anterior (TA), soleus (SOL), and medial gastrocnemius (GM). Electrode placement was conducted in accordance with the SENIAM recommendations [70] and electrodes were replaced when the skin impedance was greater than 5 kΩ. In addition, a triaxial accelerometer (Model 317A, Noraxon, Scottsdale, AZ, USA) was attached to the lateral malleolus of the affected leg for gait event detection. The electrodes were connected via preamplifiers (base gain: 500; integrated band-pass filter: 10–500 Hz) and the accelerometer directly to a telemetric system (TeleMyo 2400 G2, Noraxon USA Inc., Scottsdale, AZ, USA), whereby the transmitter unit was carried by the participants on their back. Furthermore, the EMG activity of the investigated muscles during a maximal voluntary isometric contraction (MVIC) was assessed for normalization purposes.

The following biomechanical measurements were carried out in a randomized order:

1. Isometric strength properties of the quadriceps muscle: individuals were placed in a sitting position on a previously introduced custom-built knee extension table [71]. The hip and knee joints were thereby fixed in 90° flexion and the lower end of the tibia of the affected leg was attached by a sling to a unidimensional strain gauge (KM 1500S, Megatron, Munich, Germany). Each individual was then instructed to explosively generate a maximal isometric force towards extension and to maintain this maximal force for five seconds. After a practice trial, the individuals performed two measurement trials with 15 seconds of rest in between.
2. Balance properties: individuals were instructed to assume a semi-tandem stance position (affected leg placed behind) on a force plate (type 9286BA, Kistler, Winterthur, Switzerland) and to maintain this position for 15 seconds with the gaze fixed at a point on the wall in front of them. The position of the feet was standardized with a rigid plastic frame. To ensure the individuals' safety, a physiotherapist was standing next to them during the task. After a rest of at least 30 seconds, the task was repeated one more time.
3. Gait characteristics: individuals were asked to walk a distance of 10 meters on a level surface as fast and as safely possible. Gait characteristics were thereby measured using the Locomètre® system (Satel, Toulouse, France), which was connected to the individuals' feet by two thin filaments [72]. To ensure the individuals' safety, a physiotherapist was walking next to them during the task. After a rest of at least 1 minute, the task was repeated one more time.

Strain gauge, force plate, EMG, and accelerometer signals were amplified using a custom-built universal amplifier (UMV, uk-labs, Kempen, Germany) and sampled at a rate of 1 kHz using the software package ads (version 1.12, uk-labs, Kempen, Germany), whereas the Locomètre signals were recorded without additional amplification using a software package provided by the manufacturer.

The biomechanical measurements as well as the FES-I were carried out at baseline (pre) and immediately after one series of SR-WBV training at the same day (post 1) and after the intervention period lasting two weeks (post 2), whereas the NIHSS and EBI were conducted at baseline (pre) and at the end of the intervention phase (post 2) only.

### 2.4. Intervention

Independently of the group allocation, all participants received individual conventional rehabilitation therapy (motor learning therapy, occupational, speech, and neuropsychological therapy) at every working day over a period of two weeks, resulting in ten days of therapy.). On each day of therapy as well as immediately after the baseline measurements, the individuals were additionally exposed to five one-minute sessions of SR-WBV (experimental group: frequency 5 Hz, amplitude 3 mm, noise level 4; sham group: 1 Hz, 3 mm, noise level 0) using a SR-WBV device (Zeptor med®, Frei Swiss AG, Zurich, Switzerland). The individuals in the experimental group were thereby assuming a free-standing position with the knees slightly flexed, while the individuals in the sham group were sitting on a wooden box with the legs placed on the vibration device. In addition, all individuals were asked to balance a half-filled 500 ml water bottle on a tray with their nonaffected arm during vibration exposure. After each vibration session, they were allowed to rest for one minute in a seated position.

### 2.5. Data Analysis and Outcome Measures

Several signals that were recorded during the strength and balance measurements were analyzed using the “ADS” software, whereas the gait measurements were processed using the Locomètre software and a custom-made LabVIEW program (version 11.0.1, National Instruments Corp., Austin, TX, USA).

The primary outcome parameters were defined as maximal voluntary isometric contraction (MVIC [N]) and rate of force development (RFD [N/s]), postural sway distance [32] and sway velocity [mm/s] in the mediolateral and anterior-posterior axes (calculated on the basis of the proportion of the four force sensors signals of the force plate), and gait velocity [m/s], step length [m], and stance phase duration [% of gait cycle] of the affected and unaffected legs. Secondary outcome parameters included average EMG activity [%MVIC] of several muscles during the balance task, average EMG activity [%MVIC] of several muscles during a gait cycle of the affected leg, and total scores of the NIHSS, EBI, and FES-I. For the biomechanical measurements, parameters from both trials were averaged.

### 2.6. Statistics

Based on an a priori power analysis using the software G*∗*Power (Faul et al. 2007), a theoretical sample size of N=70, was determined (repeated measures: within-between interactions ANOVA approach; effect size f=0.20, *α* error probability=0.05, power 1-*β*=0.90, groups=2, repetitions=2, correlation among repeated measures=0.5, nonsphericity correction *ε*=1, and expected dropouts=2).

Statistical analyses were based on the intention-to-treat approach (LOCF: last observation carried forward) and carried out using the software package SPSS 24 (SPSS Inc., Chicago, IL, USA). Shapiro Wilk normality tests revealed nonnormal distribution for the majority of the parameters and therefore, nonparametric procedures were applied. To investigate the short-term and long-term effects within the experimental group and sham group separately, parameters were compared between the baseline measurements (pre) and the retest measurements immediately after the first intervention (post 1) and the retest measurements after two weeks of intervention (post 2), respectively, using the Wilcoxon signed-rank test. Comparisons between the experimental group and sham group at the time points pre, post 1, and post 2 were conducted using the Mann–Whitney U test. A Bonferroni-corrected alpha-level of 0.025 was used to determine statistical significance for all tests.

## 3. Results

### 3.1. Participants

**Figure 1:**
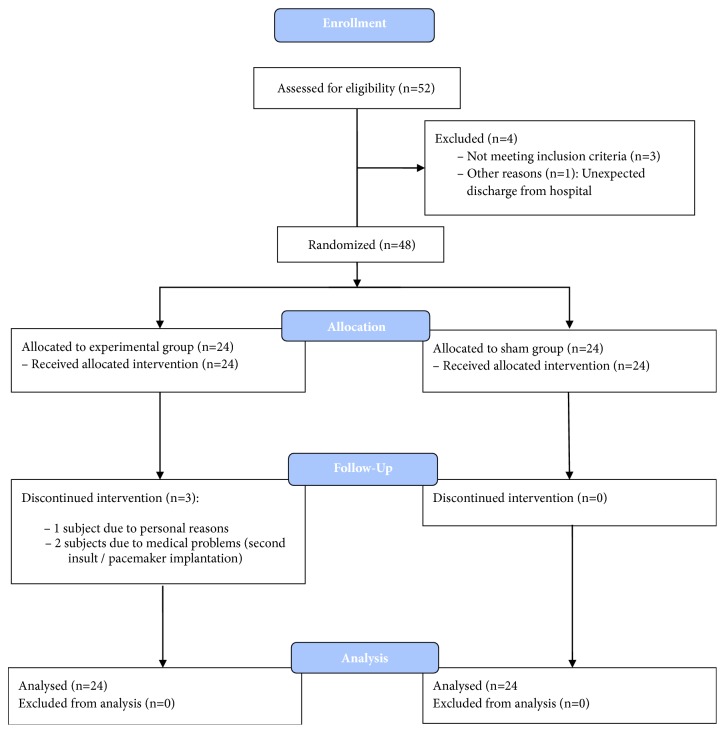
CONSORT diagram of flow of participants through the study.

The recruitment period started in June 2010 and was stopped in October 2014 due to a lack of eligible participants. Four of the 52 initially assessed participants had to be excluded due to cognitive deficits, thrombophilia, retinal hemorrhage, and discharge during intervention time (Figure 1). Forty-eight participants were finally randomly and uniformly assigned to the experimental and sham group and completed the intended treatment. Based on the intention-to-treat approach, the 3 participants that discontinued the intervention in the experimental group were also included into final analysis.

Group comparisons showed no significant differences for demographics at baseline as well as amount of completed complementary SR-WBV sessions at discharge (post 2) (Table 1).

### 3.2. Primary and Secondary Outcome Parameters

Descriptive statistics were calculated and presented as medians with the respective 25th and 75th percentiles (Table 2). No statistically significant between-group differences were found at pre, post 1, and post 2 (Table 3).

Within-group comparisons, on the other hand, revealed short-term main effects (pre to post 1) for gait velocity as well as step length and stance phase duration on the affected and unaffected sides in both groups. In the experimental group, muscle activity was found to be increased for VM during gait and balance and decreased for GM during gait. The FES-I indicated no short-term effects after one SR-WBV training session.

Long-term effects (pre to post 2) could be found by a distinct increase in isometric muscle strength (experimental group) and a reduction of sway distance (ml, ap) and sway velocity (ap) in the sham group. Both groups improved regarding gait velocity, step length, and stance phase duration of the affected and unaffected leg. Tibialis anterior activity in the sham group during gait was higher after the complete intervention period compared to baseline. Long-term effects could also be found in the total scores of the FES-I and EBI in both groups and the NIHSS in the sham group.

## 4. Discussion

This study examined the short- and long-term effects of complementary SR-WBV on balance, strength, gait, fear of falling, and performance in activities of daily life in individuals in the acute phase of stroke and TBI randomly allocated to an experimental or sham group. The results indicated no beneficial between-group effects for complementary SR-WBV on isometric quadriceps strength, static balance, and gait performance immediately after one session and after two weeks of daily training. Both groups showed comparable short- and long-term effects for gait performance, FES-I, NIHSS, and EBI. In addition, long-term effects were found for balance performance but only in the sham group.

On the one side, these findings are in line with the results of several systematic reviews and meta-analyses showing no benefits or even adverse effects of SS-WBV on muscle strength, balance, fall rate, gait performance, mobility, activity, and participation after stroke [45, 73, 74]. On the other side, the current findings deviate from those of many individual experimental studies reporting WBV-dependent functional improvements in a stroke population.

### 4.1. Short-Term Effects

Tihany et al. [38], for instance, showed significant transient improvements of isometric and eccentric maximum knee extensor strength (36.6% and 22.2%, respectively) in individuals with acute stroke after one session of WBV with a frequency of 20 Hz and an amplitude of 5 mm, which has been shown to be an effective treatment modality in healthy young adults to increase muscle strength [75, 76]. Van Nes et al. [42] provided preliminary evidence for positive short-term effects of SS-WBV on some aspects of static balance in 23 individuals with chronic stroke using an economic test setting with long rest intervals of 30 minutes in order to minimize exhaustion. Furthermore, results of an investigation on the effect of a 10-minute SS-WBV session (frequency: 12 Hz, amplitude: 4 mm) on gait performance indicated improvements in gait speed and mobility quantified by the Timed Up and Go Test [36].

These discrepancies are most likely due to several factors. The vibration intensity used in the current study (frequency: 5 Hz, amplitude: 3 mm) might have been too low in order to induce the expected effects.

In addition, the participants wore shoes to stabilize their ankle joints during SR-WBV and therefore, it cannot be excluded that the possibly insufficient impulse was additionally damped by the participants' shoes [77]. According to Freeman and Wyke [78], sensorimotor exercises are best performed without shoes to provide a maximum amount of appropriate afferent information for the sensorimotor system. Rogan et al. [79] showed that vibration training without shoes in comparison to vibration training with shoes [80] improved significantly the isometric rate of force development and increased the physical performance level in frail elderly individuals after four weeks of SR-WBV.

Furthermore, the duration of the functional measurements was about 90 minutes and included almost no resting periods. Considering the fact that individuals with stroke and TBI complain about increased mental fatigue and physical fatigability [81–85], where such an intensive testing procedure might have resulted in pronounced fatigue.

### 4.2. Long-Term Effects

Tihany et al. [41] described a significant increase in isometric and eccentric strength of the knee extensors after 4 weeks of SS-WBV with a frequency of 20 Hz and an amplitude of 1 mm, whereby the affected leg could benefit more (32.8% and 24%, respectively) than the unaffected one (10.4% and 11.6%, respectively). For eccentric strength, an increase exceeding 22% was previously considered a clinically relevant improvement for individuals with stroke [86]. Tankisheva et al. [28] reported an isometric knee extensor strength gain of 18.7% after 6 weeks SS-WBV with increasing intensity (frequency: 35-40 Hz, amplitude: 1.7-2.5 mm). Both of these studies included challenging dynamic strength exercises such as squatting during SS-WBV sessions. Furthermore, several studies showed beneficial long-term effects of 4-6 weeks SS-WBV with vibration frequencies of 15-40 Hz on balance performance in individuals with stroke [28, 39, 40, 87, 88]. Regarding gait performance, Guo et al. [89] reported improvements in gait speed after an 8-week SS-WBV training in individuals with stroke.

As for the short-term effects, it can be assumed that the vibration intensity in the current study was too low to induce detectable changes. This assumption was to some extend supported by Petit et al. [90], who compared the effects of a high frequency/high amplitude (50 Hz/4 mm) with a low-frequency/low amplitude (30 Hz/2 mm) 6-week SS-WBV intervention in young male students. They found that high frequency/high amplitude vibration training was more effective in enhancing knee extensor strength and jump performance. Interestingly, Lee [40] could improve postural control in chronic stroke survivors using a vibration plate generating horizontal vibrations at a rate of 1-3 Hz and an amplitude of 3 mm. However, placebo effects can thereby not be excluded due to a missing sham intervention.

In the current study, participants had to stand quietly with slightly flexed knees on the vibration platform, which together with the low vibration intensity might have resulted in an even weaker physiological stimulus. In addition, all participants were standing on two separately vibrating platforms. Individuals with stroke or TBI though are not always able to distribute their body weight equally over their feet and therefore, it cannot be excluded that the participants shifted their body weight mainly over the healthy leg, resulting in an insufficient stimulation of the affected leg [91, 92].

Considering that several of the above-mentioned studies included intervention periods of 4 or more weeks, the two weeks with altogether 11 SR-WBV sessions in the current study might not have been sufficient. In contrast to this assumption, however, there is evidence showing that even with higher vibrations intensities and longer treatment durations, no beneficial effects of WBV were found in postacute and chronic stroke survivors [43, 47–50]. Moreover, some of the above described beneficial effects have to be interpreted carefully, mainly due to small sample sizes (n≤20) and to the fact that WBV was administered as a supplementary treatment in the experimental group, but not in the control group [28, 38, 41].

### 4.3. Limitations

The fact that the participants were in the acute phase of stroke and TBI, during which spontaneous recovery can be observed [93–96], was considered a limitation. The current study findings are therefore not applicable individuals with chronic stroke, whereas spontaneous recoveries could have distorted the treatment effects.

Due to difficulties in finding enough eligible patients, recruitment was stopped after 4 years of intensive efforts and 48 instead of the targeted 70 participants. Consequently, statistical analyses were underpowered, which represented another limitation of this study.

## 5. Conclusions

This study evaluated the short- and long-term effects of complementary SR-WBV on muscle strength, balance, and gait performance in individuals with stroke and TBI. Complementary daily SR-WBV sessions showed no additional effects compared to a sham intervention. Future research is required to identify the potential efficacy of SR-WBV protocols in individuals with stroke or TBI, particularly with regard to intensity of the vibration parameters and the duration of the intervention considering the impaired physical and mental capability of the individuals.

## Figures and Tables

**Table 1:** Demographics of the individuals with stroke and traumatic brain injury (TBI), therapy characteristics [n, arithmetic mean (lower/upper limit 95% confidence interval), and significance test] at baseline (pre) and amount of completed complementary SR-WBV sessions at discharge (post 2).

**Variables**	**Experimental group**	**Sham group**	**Significance ** **(p)**
Participants [n (m/f)]	24 (15/9)	24 (17/7)	0.540^a^
Stroke, TBI [n, n]	20, 4	22, 2	0.383^a^
Age [years]	48.8(42.5/55.1)	46.4 (41.4/51.4)	0.364^b^
Height [m]	1.72 (1.68/1.76)	1.72 (169.4/174.5)	0.967^b^
Mass [kg]	75.1 (68.0/82.1)	76.0 (69.6/82.4)	0.804^b^

OT single [hours]	6.6 (5.5/7.7)	6.3 (5.6/7.1)	0.873^b^
OT group [hours]	5.6 (3.2/8.0)	4.1 (2.6/5.6)	0.449^b^
PT single [hours]	7.9 (6.6/9.2)	7.9 (6.9/8.9)	0.382^b^
PT group [hours]	5.7 (3.9/7.4)	6.3 (4.7/7.8)	0.253^b^
PSY single [hours]	4.4 (3.3/5.4)	4.1 (3.4/4.7)	0.881^b^
PSY group [hours]	5.7 (4.3/7.1)	6.0 (4.9/7.2)	0.670^b^
ST single [hours]	3.0 (1.0/4.9)	2.3 (1.0/3.7)	0.764^b^
ST group [hours]	0.1 (0.02/0.2)	0.3 (0.01/0.59)	0.171^b^
SR-WBV [sessions]	10.4 (10.1/10.7)	11.0 (10.1/11.3)	0.153^b^

*n*: numbers, *m*: male, *f*: female, *m*: meter, *kg*: kilogram, *OT*: occupational therapy, *PT*: physical therapy, *PSY: *psychotherapy, *ST*: speech therapy, *SR-WBV*: stochastic resonance whole body vibration, and *p*: probability; ^a^Chi square; ^b^Mann–Whitney U test.

**Table 2:** Descriptive statistics for the primary and secondary outcome parameters, presented as median with the 25^th^ and 75^th^ percentiles in brackets.

**Parameter**	**Experimental group**			**Sham group**		
	**pre**	**post 1**	**post 2**	**pre**	**post 1**	**post 2**
MVIC [N]	168.7 (266.2/314.2)	249.8 (172.0/320.4)	298.2 (170.0/340.0)	237.3 (162.8/324.7)	227.0 (151.9/334.0)	224.3 (122.4/358.3)
RFD [N/s]	2162.1 (866.1/3530.6)	1676.6 (772.8/3198.8)	1837.1 (1170.0/3128.9)	1351.6 (888.8/2536.0)	1389.3 (736.2/2935.1)	1531.3 (844.4/2661.2)

Sway distance ml [32]	9.5 (7.0/12.2)	7.6 (7.0/11.5)	8.5 (6.6/10.6)	10.0 (6.8/11.3)	8.5 (7.2/11.4)	8.2 (6.0/9.6)
Sway distance ap [32]	7.0 (5.1/9.3)	6.1 (4.6/9.6)	6.8 (4.0/9.5)	6.5 (4.9/11.0)	5.8 (4.6/9.7)	5.1 (4.3/8.4)
Sway velocity ml [mm/s]	43.7 (32.6/54.8)	37.6 (29.8/51.1)	40.8 (30.0/48.0)	32.5 (28.0/42.2)	31.2 (27.0/42.7)	31.8 (24.7/39.7)
Sway velocity ap [mm/s]	46.2 (33.0/78.2)	50.3 (31.4/71.8)	46.8 (30.7/73.0)	42.2 (29.8/65.2)	37.9 (26.6/52.0)	33.5 (29.4/52.6)

Gait velocity [m/s]	1.29 (0.90/1.64)	1.30 (0.94/1.70)	1.61 (1.22/2.06)	1.29 (0.79/1.57)	1.40 (1.01/1.70)	1.61 (1.35/1.78)
Step length al [m]	.63 (.51/.73)	.67 (.57/.77)	.71 (.59/.79)	.61 (.50/.83)	.65 (.55/.80)	.75 (.60/.91)
Step length ul [m]	.67 (.51/.74)	.65 (.52/.73)	.71 (.63/.81)	.63 (.49/.82)	.64 (.49/.80)	.74 (.58/.85)
Stance phase a [%]	53.9 (50.7/57.5)	52.8 (48.8/55.6)	51.0 (46.1/55.2)	52.7 (50.9/55.9)	52.5 (48.5/53.9)	48.2 (44.7/52.8)
Stance phase ul [%]	56.5 (50.7/62.1)	56.0 (50.5/59.1)	52.7 (49.2/55.6)	54.8 (52.4/62.0)	54.4 (52.5/61.6)	51.7 (49.9/57.0)

Balance TA [%MIVC]	15.5 (5.1/32.4)	9.8 (4.7/22.5)	11.3 (5.3/26.5)	11.6 (6.2/19.1)	9.5 (5.1/18.8)	9.6 (6.2/20.7)
Balance VM [%MIVC]	12.5 (7.8/17.0)	15.5 (8.3/23.8)	9.0 (7.0/19.6)	12.9 (7.6/31.5)	14.1 (6.4/42.4)	11.9 (7.9/22.9)
Balance VL [%MIVC]	13.2 (9.3/25.6)	17.7 (12.4/26.5)	11.6 (8.7/21.6)	21.5 (9.1/30.7)	16.1 (7.9/32.5)	16.3 (6.7/28.3)
Balance GM [%MIVC]	39.7 (28.2/74.2)	35.6 (22.4/60.9)	28.7 (20.2/57.5)	31.2 (17.5/53.5)	32.1 (15.2/52.5)	30.3 (15.7/58.4)
Balance SOL [%MIVC]	34.3 (29.6/51.7)	31.5 (24.3/54.4)	33.8 (27.4/54.1)	32.1 (25.4/64.9)	32.2 (20.8/61.0)	31.2 (24.8/52.2)

Gait TA [%MIVC]	19.2 (12.4/21.8)	16.5 (12.9/24.4)	18.5 (12.7/25.2)	14.3 (10.4/21.6)	14.1 (10.1/18.8)	18.4 (13.8/27.5)
Gait VM [%MIVC]	1.3 (8.4/15.5)	11.1 (8.6/17.4)	10.33 (8.2/16.0)	11.1 (7.4/15.9)	11.4 (7.3 (18.1)	12.2 (10.2/28.5)
Gait VL [%MIVC]	10.7 (7.2/17.4)	12.3 (8.4/16.8)	10.4 (7.3/15.0)	12.8 (7.8/16.5)	13.3 (8.9/19.5)	15.0 (10.8/22.3)
Gait GM [%MIVC]	31.8 (22.7/61.8)	35.2 (22.6/62.0)	34.7 (25.8/49.0)	26.1 (207/45.7)	26.4 (18.6/43.0)	29.2 (22.8/42.9)
Gait SOL [%MIVC]	28.8 (19.9/37.3)	29.2 (22.5/46.7)	30.8 (25.1/44.6)	30.7 (21.2/41.1)	33.1 (21.0/54.4)	36.6 (24.9/60.0)

FES-I (Score 16-64)	24 (18/30)	22 (18/28)	20 (17/24.75)	25 (20/40)	23 (18/38)	20.5 (17/28)
NIHSS (Score 0-42)	4 (3/6)	-	4 (2.25/5)	4 (3/5.75)	-	4 (2.25/5)
EBI (Score 0-64)	54.5 (43.25/59)	-	60.5 (56/62.75)	55 (46/57.5)	-	59 (55.25/63.75)

*Pre: *baseline*, post 1: *immediately after first SR-WBV intervention*, post 2*: after two weeks of SR-WBV intervention*, vs: versus, MVIC: *maximum voluntary isometric contraction*, N:* Newton, *RFD:* rate of force development, *N/s:* Newton per second, *m:* meter,* mm:* millimeter, *mm/s:* millimeter per second, *m/s:* meter per hour, *ml:* mediolateral, *ap:* anterior-posterior, *al:* affected leg, *ul:* unaffected leg, *TA:* tibialis anterior, *VM:* vastus medialis, *VL:* vastus lateralis, *GM:* gastrocnemius medialis, *SOL:* soleus, *FES-I*: Falls Efficacy Scale International, *NIHSS*: National Institute of Health Stroke Scale, and *EBI*: Extended Barthel Index.

**Table 3:** Results for the within- and between-group comparisons (Wilcoxon signed-rank and Mann–Whitney U test, respectively) of the primary and secondary outcome parameters. Statistical significance was accepted at the p≤0.025 level (Bonferroni corrected).

**Parameter**	**Within-group comparisons**				**Between-group comparisons** **Experimental group vs sham group**		
	**Short-term effects**		**Long-term effects**				
	**(pre vs post 1)**		**(pre vs post 2)**				
	**Experimental** **group**	**Sham** **group**	**Experimental** **group**	**Sham** **group**	**pre**	**post 1**	**post 2**
MVIC	.092	.543	**.022**	.761	.580	.789	.344
RFD	.034	.429	.904	.670	.566	.680	.442

Sway distance ml	.162	.989	.130	**.027**	.773	.571	.523
Sway distance ap	.932	.511	.230	**.001**	.765	.877	.464
Sway velocity ml	.511	.548	.627	.170	.063	.138	.023
Sway velocity ap	.304	.153	.211	**.010**	.452	.076	.152

Gait velocity	**.005**	**.009**	**.001**	**< .001**	.898	.924	.711
Step length al	**.001**	**< .001**	**< .001**	**< .001**	.782	.907	.270
Step length ul	**.002**	**.006**	**< .001**	**< .001**	.565	.551	.621
Stance phase al	**< .001**	**< .001**	**.002**	**.001**	.580	.924	.257
Stance phase ul	.301	.483	**.004**	**< .001**	.890	.815	.805

Balance TA	.034	.149	.420	.548	.655	.766	.916
Balance VM	**.013**	.424	.478	.116	.655	.865	.483
Balance VL	.039	.808	.247	.056	.595	.848	.782
Balance GM	**< .001**	.192	.028	.689	.181	.234	.982
Balance SOL	.241	.236	.099	.408	.566	.595	.666

Gait TA	.274	.073	.446	**.025**	.285	.100	.860
Gait VM	**.001**	.658	.243	.133	.789	.958	.125
Gait VL	.046	.809	.841	.053	.733	.774	.034
Gait GM	.601	.126	.811	.794	.308	.204	.580
Gait SOL	.970	.550	.157	.445	.521	.871	.536

FES-I	.703	.119	**< .001**	**< .001**	.415	.672	.474
NIHSS	-	-	.039	**.013**	.909	-	.983
EBI	-	-	**< .001**	**< .001**	.733	-	.852

*Pre: *baseline*, post 1: *immediately after first SR-WBV intervention*, post 2:* after two weeks of SR-WBV intervention,* vs: versus, MVIC: *maximum voluntary isometric contraction*, N:* Newton, *RFD:* rate of force development, *ml*: mediolateral, *ap:* anterior-posterior, *al*: affected leg, *ul:* unaffected leg, *TA:* tibialis anterior, *VM:* vastus medialis, *VL:* vastus lateralis, *GM:* gastrocnemius medialis, *SOL:* soleus, *FES-I*: Falls Efficacy Scale International, *NIHSS*: National Institute of Health Stroke Scale, and *EBI:* Extended Barthel Index.

## Data Availability

The datasets generated during the current study are available from the corresponding author on reasonable request.
